# Impact of Erector Spinae Plane Block on Postoperative Analgesia and Perioperative Stress Response in Sleeve Gastrectomy: A Prospective Randomized Clinical Trial

**DOI:** 10.3390/medicina62030506

**Published:** 2026-03-10

**Authors:** Kutay Barış Filazi, Nuray Altay

**Affiliations:** 1 Department of Anesthesiology and Reanimation, Ministry of Health Ağrı Education and Research Hospital, 04200 Ağrı, Türkiye; 2 Department of Anesthesiology and Reanimation, Faculty of Medicine, Harran University, Osmanbey Campus, 63300 Şanlıurfa, Türkiye

**Keywords:** sleeve gastrectomy, bariatric anesthesia, opioid-sparing analgesia, erector spinae plane block, C-reactive protein, cortisol, glucose, numerical rating scale

## Abstract

*Background and Objectives:* Effective postoperative analgesia is essential for enhanced recovery after bariatric surgery. The erector spinae plane block (ESPB) has emerged as a promising regional anesthesia technique, but its impact on postoperative pain control, opioid requirement, patient and surgeon satisfaction, and stress response in obese patients undergoing sleeve gastrectomy remains unclear. This study aimed to evaluate the effects of bilateral ESPB on postoperative analgesia requirements, pain scores, patient and surgeon satisfaction, hemodynamic stability, postoperative stress response, and perioperative hematologic and biochemical parameters in ASA II–III patients with a body mass index (BMI) > 30 undergoing sleeve gastrectomy. Study design was a prospective, randomized, single-blind clinical trial. *Materials and Methods:* After obtaining ethics committee approval (Şanlıurfa Harran University Hospital, date: 23 January 2023; decision no: HRÜ/23.02.09) and written/verbal informed consent, 60 patients aged 18–65 years, BMI > 30, ASA II–III scheduled for elective sleeve gastrectomy were included. Patients were randomized into two groups: those receiving bilateral ESPB (Group E, *n* = 30) and those without ESPB (Group C, *n* = 30). Demographic characteristics, ASA scores, comorbidities, and surgical duration were recorded. Preoperative venous samples were collected into hemogram (WBC, lymphocyte, neutrophil) and biochemistry tubes (CRP, cortisol, glucose). Standard monitoring (ECG, SpO_2_, NIBP) was applied intraoperatively, and vital parameters (HR, MAP) were recorded throughout. Postoperatively, HR, MAP, Numerical Rating Scale (NRS) scores at 0, 2, 4, 8, and 24 h, opioid requirement, patient and surgeon satisfaction (Likert scale), postoperative hemogram and biochemistry values, and side effects or complications were documented. All patients received dexketoprofen as baseline analgesia, with tramadol HCl administered as rescue analgesic. *Results:* All 60 patients completed the study. There were no statistically significant differences between the groups regarding age, BMI, or surgery duration. Comorbidities were similar between groups. Intraoperative and postoperative HR and MAP values showed no significant differences. Postoperative NRS scores at the 0, 2, 8, and 24 hours were significantly lower in Group E compared with Group C. Both patient and surgeon satisfaction scores were higher in Group E. Rescue analgesic (tramadol HCl) consumption in the postoperative ward was significantly reduced in Group E. Cortisol levels, particularly at the 24th postoperative hour, showed a significantly smaller increase in Group E, suggesting a reduced surgical stress response. No significant differences were found between the groups regarding postoperative side effects or complications. *Conclusions:* Preoperative bilateral ESPB is an effective component of multimodal analgesia in sleeve gastrectomy. The block significantly reduces postoperative pain intensity, lowers NRS scores, improves patient and surgeon satisfaction, and decreases opioid requirements. Additionally, ESPB appears to attenuate the postoperative stress response, as reflected by smaller increases in cortisol levels. These findings support the routine incorporation of ESPB in perioperative pain management strategies for gastric sleeve surgery.

## 1. Introduction

Obesity has become increasingly prevalent worldwide and is potentially becoming the most common chronic disease. According to WHO, obesity prevalence is rising in developing countries as Western dietary habits spread in developed nations [1]. Obesity is diagnosed using BMI, and in morbidly obese individuals, drug dosage is determined by adjusted body weight. Medications for surgery were adjusted to patients’ corrected weight. Due to increased risk of respiratory depression in obese patients, opioid use in postoperative pain management is restricted [2].

Sleeve gastrectomy is a recent bariatric surgical technique inducing weight loss through restrictive and endocrine mechanisms, first described by Dr. Doug Hess in 1988 [3].

Postoperative pain is a nociceptive response from surgical tissue injury, mediated by central and peripheral sensitization [4]. About 30–75% of patients experience moderate-to-severe post-surgical pain [5]. Effective multimodal pain management is essential for bariatric procedures, reducing opioid requirements and decreasing adverse effects and pulmonary complications [6,7].

Regional anesthesia can be safely used in morbidly obese patients, though increased adipose tissue makes anatomical landmark identification challenging. When opioids are added to local anesthetics, respiratory depression risk must be considered [2].

The Erector Spinae Plane Block (ESPB) is an interfacial block performed paraspinally, where local anesthetic spreads across vertebral levels, providing somatic and visceral analgesia [8]. This ultrasound-guided technique manages acute and chronic pain during various surgeries. ESPB can be administered preoperatively without sedation as single-shot or continuous infusion. It was first reported in 2016 for treating thoracic neuropathic pain [9].

The block has been successfully used in Nuss procedure, thoracotomy, nephrolithotomy, hernia repair, and lumbar fusions [10,11,12]. Studies show surgical stress response involves metabolic, endocrine, immunological, and hematological factors, including glucose, lymphocytes, neutrophils, cortisol, and CRP levels [13].

This study evaluated ESPB’s effect on postoperative analgesic requirements and stress response in sleeve gastrectomy patients. The primary outcome of this study was postoperative pain intensity assessed by the Numerical Rating Scale (NRS) at 24 h. Secondary outcomes included NRS scores at other postoperative time points, total postoperative opioid consumption, patient and surgeon satisfaction, perioperative hemodynamic parameters, and perioperative stress response assessed by laboratory markers.

## 2. Materials and Methods

### 2.1. Study Design

**Figure 1:**
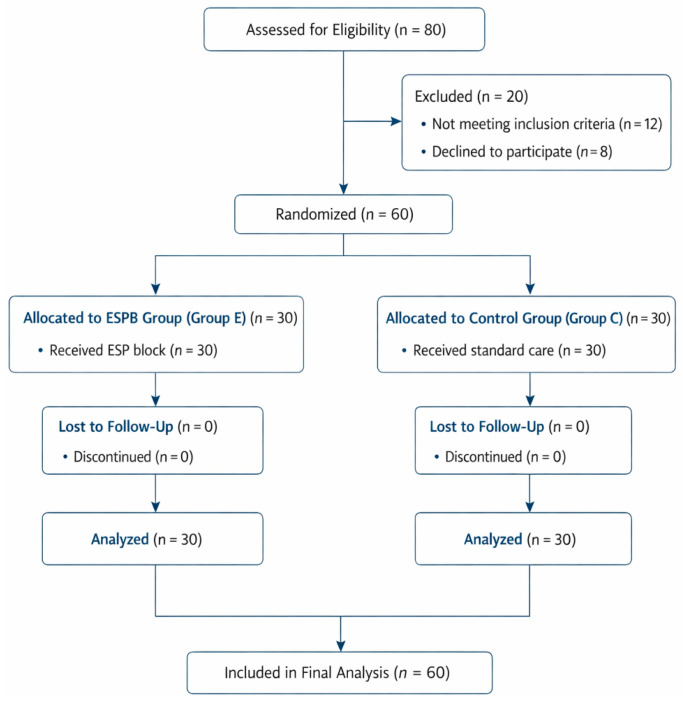
CONSORT flow diagram of patient enrollment and randomization.

This study was conducted with the approval of the Ethics Committee of Şanlıurfa Harran University Hospital Clinical Research Board (Date: 23 January 2023; Approval No: HRÜ/23.02.09), this trial was registered in ClinicalTrials.gov (identifier: NCT07431905) and was conducted in accordance with the principles of the Declaration of Helsinki. Although the study was prospectively designed and conducted in accordance with ethical approval, registration in ClinicalTrials.gov was completed after the initiation of patient recruitment due to administrative and technical delays. No changes were made to the study protocol, outcomes, or analysis plan after trial commencement. Written and verbal informed consent was obtained from all participants. The study was designed as a prospective, randomized, and single-blind trial including 60 volunteer patients aged 18–65 years, classified as ASA II–III, with a body mass index (BMI) greater than 30, who were scheduled to undergo elective sleeve gastrectomy under general anesthesia in the Department of General Surgery. This study adhered to the CONSORT (Consolidated Standards of Reporting Trials) guidelines throughout its design, implementation, and reporting (Figure 1).

### 2.2. Sample Size and Power Calculation

Although an a priori sample size calculation was not performed, a post hoc power analysis was conducted based on the primary outcome (24 h NRS score). Based on the observed difference between groups (mean ± SD: 1.67 ± 1.40 vs. 3.03 ± 1.35; *n* = 30 per group), the achieved power was 96.5% for a two-sided independent samples *t*-test at α = 0.05 (Cohen’s d = 0.99).

### 2.3. Participants

Patients aged 18–65 years with ASA physical statuses II–III and BMI > 30 who received or did not receive an erector spinae plane (ESP) block for preoperative analgesia were included. Exclusion criteria were patients < 18 or >65 years, ASA I, IV, or V classification, BMI < 30, contraindications to ESPB, and renal or hepatic insufficiency. All patients were informed about the study preoperatively, and consent was obtained. Participants were instructed on the analgesic method and visual analog scale (VAS) scoring system during evaluation.

### 2.4. Randomization and Blinding

Randomization was performed using a computer-generated random sequence. Allocation concealment was ensured using sequentially numbered, sealed, opaque envelopes prepared by an independent researcher prior to patient enrollment. Envelopes were opened only after the patient arrived in the preoperative area and provided informed consent. Patients were blinded to group allocation. Postoperative pain assessment, satisfaction scoring, and data collection were performed by an investigator blinded to group assignment. Surgeons were not informed about group allocation during postoperative assessment. The anesthesiologist performing the erector spinae plane block was necessarily aware of group assignment due to the nature of the intervention. Data analysis was conducted by an independent statistician who was blinded to group identity. Patients were divided into two groups: erector spinae plane block (Group E, *n* = 30) and control (Group C, *n* = 30). Demographic data, surgery duration, ASA physical status, and comorbidities were recorded. Venous blood samples were collected preoperatively for complete blood count and biochemical analysis, including white blood cell, lymphocyte, and neutrophil counts, as well as C-reactive protein, cortisol, and glucose levels. Patients were monitored before anesthesia induction using electrocardiography, pulse oximetry (SpO_2_), and noninvasive blood pressure monitoring. Intraoperative vital parameters were continuously recorded. Postoperatively, heart rate, mean arterial pressure, Numerical Rating Scale (NRS) pain scores, patient and surgeon satisfaction assessed using a Likert scale, total opioid consumption, preoperative and postoperative 24 h hematologic and biochemical parameters, and perioperative complications were documented.

### 2.5. Anesthetic Management and ESPB Protocol

Patients in Group E were transferred to the preoperative area 30 min before surgery for erector spinae plane block. The block was performed under ultrasound guidance (Esaote MyLab™ X7 ultrasound system, Esaote S.p.A., Genoa, Italy) by the investigator and an experienced anesthesiologist who had performed the block at least 20 times. At the T7–T8 vertebral levels, with the patient prone, the skin was sterilized using 10% povidone-iodine. A linear ultrasound probe was placed to visualize the spinous process and moved 3 cm laterally on each side. After identifying the erector spinae muscles, psoas muscle, and transverse process, the needle insertion point was determined. A 22-gauge, 100 mm peripheral nerve block needle Stimuplex® Ultra 360 (B. Braun Melsungen AG, Melsungen, Germany) was advanced until contacting the transverse process. After withdrawal, 20 mL of local anesthetic mixture (6 mL of 2% lidocaine, 10 mL of 0.25% bupivacaine, 4 mL normal saline) was injected into the fascial plane. Thirty minutes later, dermatomal mapping was performed using cold–warm discrimination, and patients were transferred to the operating table. Routine monitoring, including ECG, NIBP, and SpO_2_, was performed. General anesthesia was induced with propofol (2–3 mg/kg), remifentanil (1 µg/kg), and rocuronium (0.6 mg/kg). After muscle relaxation, patients were orotracheally intubated. Anesthesia was maintained with 50% oxygen–air mixture at 2 L/min flow and sevoflurane at 3%. All patients received standardized prophylactic antiemetic therapy consisting of intravenous ondansetron at the end of surgery.

### 2.6. Patients Who Did Not Receive Any Regional Block (Group C) and Analgesic Protocol

Patients in Group C did not receive any regional block. At the conclusion of surgery, all patients were administered 100 mg of intravenous (IV) tramadol. Neuromuscular blockade was reversed in both groups using Sugammadex at a dose of 2 mg/kg. Demographic data and intraoperative vital signs were recorded at baseline (defined as the start of surgery, 0 min) and subsequently at 15 min intervals throughout the procedure. All patients received a standardized baseline analgesic regimen consisting of 100 mg intravenous tramadol administered at the end of surgery. Postoperative opioid consumption refers exclusively to additional rescue tramadol administered in the postoperative period. Rescue analgesia was provided as intravenous tramadol on patient request or when NRS ≥ 4. Intraoperative opioid administration and the routine end-of-surgery tramadol dose were not included in postoperative opioid consumption analyses.

### 2.7. Outcome Measures

For postoperative care, patients were transferred to recovery, and initial numerical rating scale (NRS) pain scores were recorded. Patients were discharged to the surgical ward once oriented, cooperative, hemodynamically stable, and had achieved a Modified Aldrete Score of 9. Blood samples were collected preoperatively and at 24 h postoperatively in purple-capped tubes for complete blood count and yellow-capped tubes for biochemical analysis. Hematological parameters (WBC, lymphocytes, neutrophils) and biochemical parameters (CRP, cortisol, glucose) were recorded. Serum cortisol concentrations were measured and reported in mg/dL according to the laboratory reference standards of our institution. Perioperative serum cortisol level was predefined as a secondary outcome reflecting the surgical stress response. Blood samples were obtained preoperatively (baseline) and at postoperative 24 h. The primary outcome of this study was postoperative pain intensity assessed using the Numerical Rating Scale (NRS) at 24 h. Secondary outcomes included NRS scores at other postoperative time points, total postoperative opioid consumption, patient and surgeon satisfaction scores, perioperative hemodynamic parameters, and perioperative stress response assessed by cortisol levels. Perioperative cortisol level was predefined as a secondary outcome reflecting surgical stress response. NRS pain scores were documented at 0, 2, 4, 8, and 24 h postoperatively. Patients received 50 mg dexketoprofen twice daily post-surgery. Total Tramadol HCl consumption, vital signs, nausea, vomiting, and other complaints were recorded. At 24 h postoperatively, patient and surgeon satisfaction were assessed using a 5-point Likert scale (1 = not satisfied at all; 2 = slightly satisfied; 3 = moderately satisfied; 4 = satisfied; 5 = very satisfied).

### 2.8. Statistical Analysis

Statistical analyses were performed using the Statistical Package for the Social Sciences (SPSS) version 25.0 (IBM Corp., Armonk, NY, USA). Continuous variables are presented as mean ± standard deviation or median (interquartile range), as appropriate. Categorical variables are expressed as numbers and percentages. Normality of data distribution was assessed using the Shapiro–Wilk test and visual inspection of histograms. Baseline demographic and clinical characteristics were compared between groups using the independent samples t-test or Mann–Whitney U test for continuous variables, and the chi-square test or Fisher’s exact test for categorical variables, as appropriate. Repeated-measures outcomes, including postoperative Numerical Rating Scale (NRS) pain scores, hemodynamic parameters (heart rate and mean arterial pressure), and postoperative opioid consumption over time, were analyzed using linear mixed-effects models. These models included fixed effects for group, time, and the group × time interaction, with a random intercept for each subject to account for within-subject correlations across repeated measurements. Post hoc pairwise comparisons were adjusted for multiple testing using the Bonferroni correction. Ordinal outcomes, including Numerical Rating Scale (NRS) pain scores and patient and surgeon satisfaction scores measured on a 5-point Likert scale, were analyzed using the Mann–Whitney U test. Perioperative cortisol levels demonstrated a non-normal distribution and are presented as median (interquartile range). Between-group comparisons were performed using the Mann–Whitney U test. Postoperative opioid consumption demonstrated a skewed distribution with a high proportion of zero values and was therefore analyzed using non-parametric methods. In addition, postoperative cortisol levels were analyzed using analysis of covariance (ANCOVA), with baseline cortisol values included as a covariate to account for baseline differences. A *p*-value < 0.05 was considered statistically significant for all analyses.

## 3. Results

### 3.1. Baseline Demographic and Clinical Characteristics of the Study Population

This prospective trial included sixty patients (aged 18–65 years, ASA II–III) scheduled for surgery, randomly assigned into two groups of 30 patients using sealed envelopes. Group E received bilateral ultrasound-guided ESPB, while Group C served as control without ESPB. Demographic data, surgery duration, ASA classification, and comorbidities were recorded (Table 1). Baseline demographic and clinical characteristics were comparable between the two groups, with no statistically significant differences observed (*p* > 0.05 for all). Patients were monitored using ECG, SpO_2_, and NIBP before anesthesia induction, with intraoperative vital parameters documented. Postoperatively, heart rate, MAP, NRS scores, patient/surgeon satisfaction, opioid consumption, hematological parameters (WBC, lymphocytes, neutrophils), biochemical parameters (CRP, glucose, cortisol), and complications were recorded. Dermatomal sensory changes were assessed qualitatively but not quantified for statistical analysis (Table 1).

### 3.2. Effect of ESPB Timing on Intraoperative and Postoperative Hemodynamic Profiles

Intraoperative heart rates were measured and recorded at 0, 5, 15, 30, 45, and 60 min in both Group E and Group C. No significant differences were observed between the groups at any time point (*p* > 0.05). Similarly, intraoperative mean arterial pressures were recorded at the same time intervals for both groups, and no significant intergroup differences were detected (*p* > 0.05).

Postoperatively, heart rates and mean arterial pressures were recorded at 0, 2, 4, 8, and 24 h in both groups. No statistically significant differences were observed between Group E and Group C for either parameter at any postoperative time point (*p* > 0.05).

### 3.3. Effect of ESPB Timing on Postoperative Pain Intensity: NRS Score Trajectory

**Figure 2:**
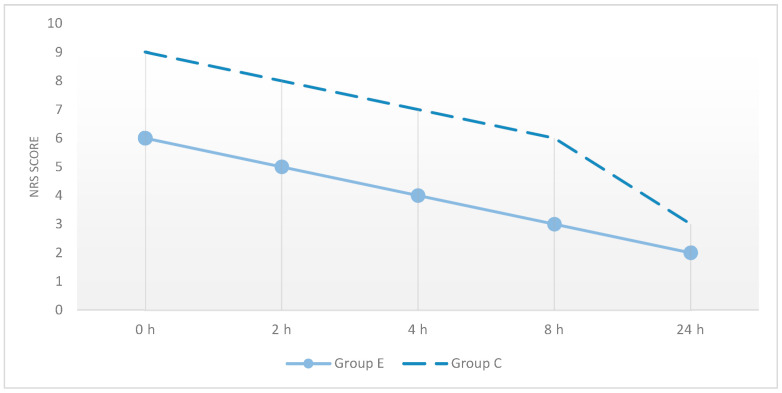
Postoperative NRS pain scores at different time points in the ESPB and control groups.

Analysis of postoperative numerical rating scale (NRS) scores revealed significant differences between Group E and Group C at 0, 2, 4, 8, and 24 h. At each time point, patients in Group E demonstrated significantly lower NRS scores compared to Group C (*p* < 0.05) (Table 2, Figure 2).

### 3.4. Impact of ESPB Administration Timing on 24-Hour Patient Satisfaction

In the assessment of patient satisfaction using the Likert scale, the mean score in Group E was 4.57 ± 0.26 (range 3–5), whereas in Group C, it was 3.07 ± 0.91 (range 1–4). The difference between the groups was statistically significant (*p* < 0.001), indicating a marked increase in patient satisfaction in Group E. Similarly, in the evaluation of surgeon satisfaction, the mean Likert score was 4.80 ± 0.41 (range 4–5) in Group E and 3.60 ± 0.77 (range 2–5) in Group C. A significant difference was also observed between the groups (*p* < 0.001), demonstrating a substantial improvement in surgeon satisfaction in Group E (Table 3).

### 3.5. Effect of ESPB Timing on the Incidence and Dose of Rescue Opioid Use

**Figure 3:**
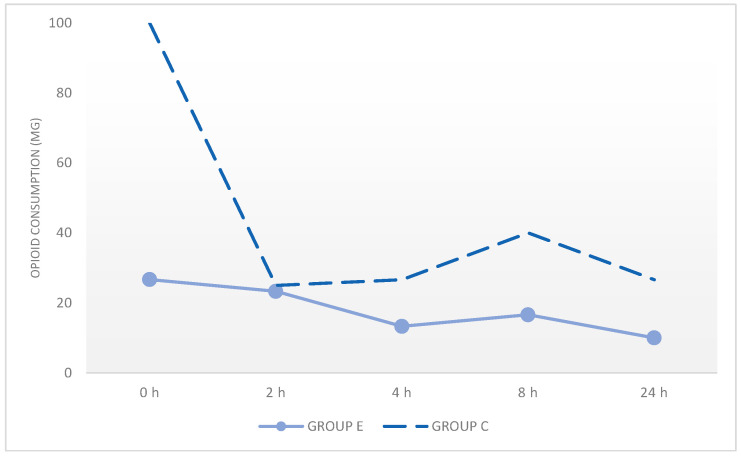
The amount of opioid use in the postoperative period between groups.

ESPB administration reduced postoperative pain and enhanced comfort. Postoperative rescue opioid consumption, defined as additional tramadol administered after surgery excluding the routine end-of-surgery dose, was lower in Group E compared with Group C. These findings decrease costs and improve patient comfort (Table 4, Figure 3).

### 3.6. Effect of ESPB Timing on Preoperative and Postoperative Hematological Parameters

Blood samples were collected preoperatively and postoperatively from both groups to evaluate hematological parameters (WBC, lymphocytes, neutrophils) and surgical stress markers in the blood (CRP, glucose, cortisol). As shown in the table, a significant difference was observed only in preoperative and postoperative cortisol levels between the groups (*p* < 0.05), whereas no significant differences were detected for the other parameters (*p* > 0.05) (Table 5).

**Figure 4:**
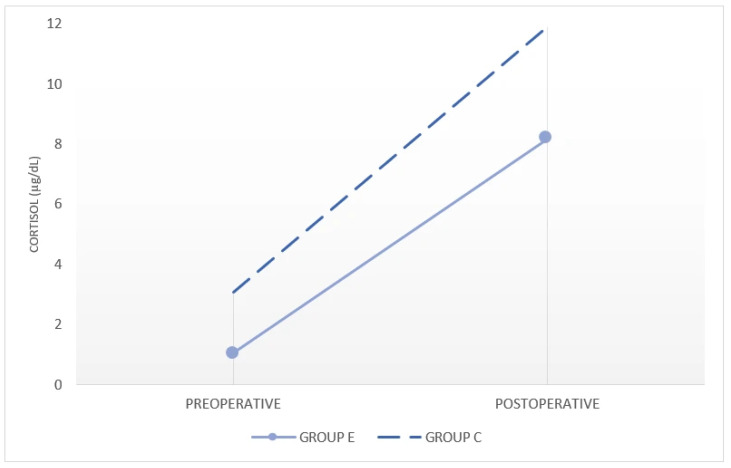
Preoperative and postoperative cortisol values between groups.

Perioperative cortisol levels demonstrated a non-normal distribution and are therefore presented as median (interquartile range). Preoperative cortisol levels were comparable between groups. Although baseline cortisol levels were slightly higher in Group C, postoperative cortisol levels remained significantly lower in Group E after adjustment for baseline values (Table 6, Figure 4). 

### 3.7. Adverse Events

Postoperative adverse events were assessed in both groups. Postoperative nausea and vomiting (PONV) were defined as the presence of any subjective complaint of nausea or at least one episode of vomiting during the first 24 postoperative hours. Using this broad definition, PONV was recorded in 29 of 30 patients (96.7%) in Group E and in 29 of 30 patients (96.7%) in Group C, with no statistically significant difference between groups (*p* > 0.05). No other serious adverse events related to the erector spinae plane block were observed.

## 4. Discussion

This study evaluated the effects of preoperative erector spinae plane block (ESPB) on perioperative hemodynamics, postoperative analgesic requirements, patient and surgeon satisfaction, hematological parameters, and stress response in ASA II–III patients with a BMI > 30 undergoing sleeve gastrectomy under general anesthesia. Demographic characteristics were generally comparable between groups; although patients in Group E were slightly older and had lower BMI values, ASA classification and comorbidities were similar. Operating time tended to be shorter in Group C, but this difference did not reach statistical significance. No significant intergroup differences were observed in intraoperative or postoperative heart rate or mean arterial pressure. The use of ESPB resulted in significantly lower postoperative pain scores, reduced analgesic consumption, higher patient satisfaction, and attenuated perioperative cortisol levels. These findings support the role of ESPB as an effective component of multimodal analgesia in bariatric surgery.

Higher preoperative cortisol levels observed in Group C may be related to their higher BMI values, suggesting a potential association between obesity and cortisol elevation. Previous data from the English Longitudinal Study of Ageing demonstrated significantly higher cortisol concentrations in obese individuals, which were associated with long-term obesity maintenance [14]. Similarly, Chan et al. reported elevated cortisol levels in obese adults, with a positive correlation between cortisol concentration and BMI [15]. Although absolute postoperative cortisol levels were lower in Group E, the relative increase appeared more pronounced due to lower baseline values.

Sleeve gastrectomy is a widely performed bariatric procedure that induces weight loss through restrictive and endocrine mechanisms [3]. Effective multimodal postoperative analgesia following bariatric surgery reduces opioid consumption and opioid-related adverse effects, thereby lowering the risk of pulmonary complications and enhancing recovery [16,17]. Adequate postoperative analgesia may reduce surgical stress response and enhance recovery [6,7]. The effective use of regional analgesic techniques strengthens multimodal analgesia and reduces postoperative analgesic needs.

Effective postoperative pain management is a cornerstone of anesthetic practice. Inadequate analgesia may result in neurohormonal activation and central sensitization, increasing the risk of chronic pain development. Adequate pain control improves patient satisfaction, reduces postoperative complications, and may shorten hospital stay [18]. Ultrasound-guided peripheral nerve blocks are safe and effective components of multimodal analgesia; however, despite their increasing use, a substantial proportion of patients continue to experience moderate-to-severe postoperative pain [5,19]. Uncontrolled pain has been associated with adverse cardiovascular events, respiratory complications, reduced mobility, and an increased risk of thromboembolic events [20].

Therefore, in the present study, a preoperative erector spinae plane block (ESPB) was administered to patients undergoing sleeve gastrectomy to enhance postoperative analgesia. The erector spinae plane block, first described by Forero et al. in 2016 for the treatment of neuropathic pain, has gained increasing attention as an effective technique for acute perioperative pain management [9]. When performed at lower thoracic levels (T7–T9), ESPB provides analgesia suitable for laparoscopic abdominal procedures by covering cervical, thoracic, and lumbar dermatomes [21,22,23].

Several alternative analgesic techniques, including transversus abdominis plane (TAP) block and port-site local anesthetic infiltration, have been utilized for postoperative pain management in bariatric surgery. These approaches primarily target somatic pain arising from abdominal wall incisions and have been shown to reduce early postoperative pain and postoperative rescue opioid requirement [24].

However, bariatric procedures such as sleeve gastrectomy involve both somatic and visceral pain components. While TAP block and local infiltration may effectively address incisional pain, their influence on visceral nociception and the perioperative stress response appears limited [25]. In contrast, ESPB may offer broader analgesic coverage through potential paravertebral spread, allowing modulation of both somatic and visceral nociceptive pathways [9]. This broader effect may explain the observed improvements in postoperative pain scores, opioid consumption, and stress hormone attenuation in the present study.

Both clinical and cadaveric studies have investigated the mechanism of action of ESPB and the spread of local anesthetics. Forero et al. demonstrated that local anesthetic can spread to the paravertebral space and reach the ventral rami of spinal nerves via the costotransverse foramina [9]. Similarly, Choi et al. reported paravertebral, intervertebral foraminal, and spinal nerve spread of local anesthetic in cadaveric models [26]. Additional cadaveric studies have shown staining of both dorsal and ventral rami following ESPB; however, the presence of a consistent open channel and the precise role of the costotransverse foramina as conduits remain incompletely understood [27,28]. Beyond macroscopic injectate spread, emerging mechanistic evidence suggests that the analgesic effects of interfascial plane blocks may also involve tissue-level diffusion of lipophilic local anesthetics across fascial planes and muscle tissue. This diffusion may facilitate secondary neural engagement beyond the immediately visualized sonographic compartment, potentially contributing to broader segmental analgesia than expected based on imaging alone. Although the present study did not assess local anesthetic spread using imaging techniques, these mechanistic considerations are consistent with the observed reductions in postoperative pain scores and opioid consumption following ESPB administration [29]. This concept may help reconcile discrepancies between anatomical spread observed in cadaveric or imaging studies and the clinical analgesic efficacy reported after ESPB.

Clinical studies further support the analgesic efficacy of ESPB. Aksu et al. evaluated the effects of ESPB on postoperative analgesia and opioid consumption in patients undergoing breast surgery and demonstrated significantly reduced morphine requirements at 6, 12, and 24 h postoperatively, with an approximately 75% reduction in total opioid consumption over 24 h [30]. Although no significant differences in NRS scores were observed, ESPB was associated with reduced opioid-related adverse effects and increased patient satisfaction [31]. Consistent with previous reports, postoperative NRS scores at 0, 2, 4, 8, and 24 h were significantly lower in Group E than in Group C in the present study.

ESPB was associated with reduced postoperative analgesic requirements and higher patient satisfaction. Supporting these findings, Eskin et al. reported increased patient satisfaction following ESPB and modified thoracolumbar paravertebral block in lumbar disc herniation surgery [32], while Murouchi et al. found comparable satisfaction between paravertebral and retrolaminar blocks in breast surgery [33]. The higher patient satisfaction observed in Group E in our study is likely attributable to improved analgesia and reduced opioid consumption.

Özcan et al. evaluated the effects of epidural anesthesia on the surgical stress response by comparing postoperative plasma cortisol and glucose concentrations and reported significantly lower levels at 4 h postoperatively in the epidural anesthesia group [34]. Similarly, Kendrisic et al. assessed serum cortisol levels in patients undergoing hip arthroplasty under different anesthetic techniques, including general, epidural, spinal, and peripheral nerve block anesthesia, and demonstrated significantly lower cortisol concentrations at 4 h postoperatively in regional anesthesia groups, with persistently lower levels at 12 h in patients receiving continuous catheter analgesia [35]. Consistent with these findings, the present study demonstrated significantly smaller perioperative cortisol increases in Group E compared to Group C.

The observed reduction in perioperative cortisol levels in patients receiving the erector spinae plane block may be explained by attenuation of nociceptive input to central stress pathways. Surgical trauma and postoperative pain activate afferent nociceptive signaling, leading to stimulation of the hypothalamic–pituitary–adrenal (HPA) axis and subsequent systemic cortisol release. Effective regional analgesia has been shown to blunt this neuroendocrine stress response by reducing pain-related afferent transmission to the hypothalamus [36].

By providing effective thoracic and upper abdominal analgesia, ESPB may reduce afferent nociceptive input at the spinal level, thereby suppressing activation of the hypothalamic–pituitary–adrenal (HPA) axis. Anatomical and imaging studies have demonstrated that ESPB may allow paravertebral and epidural spread of local anesthetic, facilitating modulation of both somatic and visceral pain pathways [9]. This broader analgesic coverage may account for the consistently lower perioperative cortisol levels observed in Group E in the present study.

Attenuation of the perioperative stress response has been associated with improved metabolic stability, reduced insulin resistance, and enhanced postoperative recovery, which may be particularly relevant in obese patients undergoing bariatric surgery [25,36]. Therefore, the reduction in cortisol levels observed in our study may reflect not only effective analgesia but also a more favorable physiological stress profile. Although cortisol reduction is not a direct clinical endpoint, it represents an objective marker of surgical stress, and its attenuation may contribute to improved pain control, reduced opioid consumption, and enhanced early postoperative recovery.

C-reactive protein (CRP) is a marker of the acute-phase inflammatory response to surgical trauma, with levels typically increasing within 12 h, peaking at 48–72 h, and returning to baseline within one week [37]. Bagry et al. found continuous lumbar plexus and sciatic nerve blocks were associated with lower serum CRP levels and leukocyte counts, indicating reduced inflammation [38]. In the present study, no significant differences were observed between the ESPB and control groups with respect to postoperative CRP and glucose levels.

The high incidence of PONV observed in both groups likely reflects the broad definition used in this study, which included any subjective complaint of nausea, rather than clinically significant vomiting requiring rescue antiemetic therapy.

### Limitations

This study has several limitations that should be acknowledged. First, the relatively small sample size and the evaluation of hematological and biochemical parameters limited to the first 24 postoperative hours may restrict the generalizability of the findings and preclude assessment of long-term outcomes. Consequently, the sustained postoperative analgesic efficacy of ESPB after hospital discharge, its long-term impact on patient satisfaction, and its potential influence on the development of chronic pain could not be evaluated. Although an a priori sample size calculation was not performed, post hoc power analysis demonstrated that the study was adequately powered to detect the observed difference in 24 h NRS scores. Future studies with larger sample sizes and extended follow-up periods are warranted to further clarify the long-term clinical implications of ESPB. Given the evaluation of multiple secondary and exploratory outcomes, the risk of type I error due to multiplicity cannot be excluded. Therefore, secondary outcomes should be interpreted as exploratory and hypothesis-generating rather than confirmatory. Complete double blinding was not feasible because the anesthesiologist performing the block was aware of group allocation. This may have introduced performance bias, particularly for subjective outcomes such as pain scores and satisfaction. However, patient blinding and blinded outcome assessment were used to minimize this risk.

## 5. Conclusions

Multimodal analgesic strategies have become vital after bariatric surgery. These strategies reduce opioid consumption in obese patients, minimizing drug-related effects and pulmonary complications. In morbidly obese patients, increased adipose tissue poses technical challenges for regional anesthesia techniques. Anatomical landmark identification may be difficult, requiring specialized needles. Ultrasound-guided (USG) regional anesthesia has provided a safe, effective approach for obese patients.

The erector spinae plane block (ESPB) is a promising technique for reducing postoperative pain. In our study, ESPB was associated with decreased pain, reduced NRS scores, increased patient satisfaction, and lower opioid consumption in patients who underwent sleeve gastrectomy. Furthermore, ESPB attenuated the postoperative increase in cortisol levels, a marker of surgical stress response.

In conclusion, preoperative US-guided ESPB is effective and reliable for obese patients undergoing sleeve gastrectomy under general anesthesia and may serve as a component of multimodal postoperative analgesia. However, large-scale prospective studies are needed to evaluate the effects of ESPB on multimodal analgesia and surgical stress response.

## Figures and Tables

**Table 1:** Baseline demographic and clinical characteristics stratified by study group.

		Group E (*n* = 30)	Group C (*n* = 30)	*p* Value
Age (years)	Mean ± SD Min–Max	37.57 ± 10.78 17–63	35.63 ± 12.87 18–63	0.531
Sex	Female	18 (60.00)	19 (63.33)	
	Male	12 (40.00)	11 (36.67)	
Weight (kg)	Mean ± SD Min–Max	123.29 ± 18.63 91–165	131.36 ± 24.91 92–208	0.395
Height (cm)	Mean ± SD Min–Max	164.33 ± 9.62 147–186	164.13 ± 6.50 153–179	0.625
BMI (kg/m^2^)	Mean ± SD Min–Max	45.30 ± 4.55 36.80–54.77	48.72 ± 8.51 36.05–71.97	0.057
Operation Time (minutes)	Mean ± SD Min–Max	89.33 ± 29.99 60–185	77.83 ± 12.30 50–100	0.059
ASA	II	22 (73.33)	22 (73.33)	
	III	8 (26.67)	8 (26.67)	

**Table 2:** Temporal comparison of postoperative NRS pain scores by ESPB timing.

Postoperative Time		Group E (*n* = 30)	Group C (*n* = 30)	*p* Value
NRS 0 h	Mean ± SD Min–Max	6.27 ± 2.82 0–10	8.80 ± 1.47 5–10	*p* < 0.001
NRS 2 h	Mean ± SD Min–Max	5.10 ± 2.45 0–9	8.13 ± 1.63 5–10	*p* < 0.001
NRS 4 h	Mean ± SD Min–Max	3.77 ± 2.03 0–8	6.77 ± 1.55 4–9	*p* < 0.001
NRS 8 h	Mean ± SD Min–Max	2.93 ± 1.76 0–7	5.70 ± 1.62 2–8	*p* < 0.001
NRS 24 h	Mean ± SD Min–Max	1.67 ± 1.40 0–5	3.03 ± 1.35 1–5	*p* < 0.001

**Table 3:** Likert-based assessment of patient satisfaction 24 h after ESPB.

		Group E (*n* = 30)	Group C (*n* = 30)	*p* Value
Patient	Mean ± SD Min–Max	4.57 ± 0.26 3–5	3.07 ± 0.91 1–4	*p* < 0.001
Surgeon	Mean ± SD Min–Max	4.80 ± 0.41 4–5	3.60 ± 0.77 2–5	*p* < 0.001

**Table 4:** Postoperative rescue opioid consumption (intravenous tramadol, mg) excluding routine end-of-surgery dosing (mean ± SD).

		Group E (*n* = 30)	Group C (*n* = 30)	*p* Value
Amount of Opioid Used (mg) Postoperative 0 h	Mean ± SD Min–Max	26.67 ± 44.98 0–100	100.00 ± 0.00 100–100	*p* < 0.001
Amount of Opioid Used (mg) Postoperative 2 h	Mean ± SD Min–Max	23.33 ± 43.02 0–100	25.00 ± 43.05 0–100	0.881
Amount of Opioid Used (mg) Postoperative 4 h	Mean ± SD Min–Max	13.33 ± 34.58 0–100	26.67 ± 44.98 0–100	0.203
Amount of Opioid Used (mg) Postoperative 8 h	Mean ± SD Min–Max	16.67 ± 37.91 0–100	40.00 ± 49.83 0–100	0.046
Amount of Opioid Used (mg) Postoperative 24 h	Mean ± SD Min–Max	10.00 ± 30.51 0–100	26.67 ± 44.98 0–100	0.098

Postoperative 0 h represents rescue tramadol administered in the recovery unit. Routine intravenous tramadol administered at the end of surgery was not included in the analysis.

**Table 5:** Effect of ESPB Timing on Preoperative and Postoperative Hematological Parameters (mean ± SD).

		Group E (*n* = 30)	Group C (*n* = 30)	*p* Value
Preoperative WBC (10^3^/mm^3^)	Mean ± SD Min–Max	9.82 ± 2.58 4.85–16.50	9.43 ± 1.84 5.55–14.61	0.498
Preoperative LYMPHOCYTE (%)	Mean ± SD Min–Max	31.28 ± 8.80 8.73–48.32	30.02 ± 9.19 4.17–55.22	0.591
Preoperative LYMPHOCYTE (10^3^/mm^3^)	Mean ± SD Min–Max	3.02 ± 0.92 0.42–4.56	2.75 ± 0.81 0.45–4.23	0.230
Preoperative NEUTROPHIL (%)	Mean ± SD Min–Max	58.44 ± 9.82 41.30–81.56	60.71 ± 11.39 33.06–94.62	0.410
Preoperative NEUTROPHIL (10^3^/mm^3^)	Mean ± SD Min–Max	5.79 ± 2.09 2.65–12.70	5.88 ± 1.64 2.88–10.22	0.844
Preoperative CRP (mg/L)	Mean ± SD Min–Max	0.72 ± 0.68 0.05–2.94	0.82 ± 0.75 0.09–3.15	0.594
Preoperative GLUCOSE (mg/dL)	Mean ± SD Min–Max	117.13 ± 41.04 81.00–247.00	120.00 ± 57.69 61.00–323.00	0.825
Postoperative 24 h WBC (10^3^/mm^3^)	Mean ± SD Min–Max	12.27 ± 3.15 6.40–21.80	12.15 ± 2.60 7.90–18.88	0.875
Postoperative 24 h LYMPHOCYTE (%)	Mean ± SD Min–Max	17.20 ± 6.62 6.95–31.50	19.19 ± 11.84 4.96–70.30	0.425
Postoperative 24 h LYMPHOCYTE (10^3^/mm^3^)	Mean ± SD Min–Max	1.98 ± 0.65 0.30–3.21	1.90 ± 0.55 0.66–3.12	0.607
Postoperative 24 h NEUTROPHIL (%)	Mean ± SD Min–Max	74.81 ± 8.40 54.30–88.70	74.51 ± 8.84 59.90–91.17	0.894
Postoperative 24 h NEUTROPHIL (10^3^/mm^3^)	Mean ± SD Min–Max	9.22 ± 3.03 4.45–19.20	9.11 ± 2.80 4.67–15.90	0.895
Postoperative 24 h CRP (mg/L)	Mean ± SD Min–Max	2.49 ± 1.70 0.50–8.36	2.47 ± 1.88 0.60–9.19	0.954
Postoperative 24 h GLUCOSE (mg/dL)	Mean ± SD Min–Max	141.97 ± 36.98 80.00–219.00	133.03 ± 44.66 75.00–279.00	0.402

**Table 6:** Effect of ESPB Timing on Preoperative and Postoperative Cortisol Parameters.

		Group E (*n* = 30)	Group C (*n* = 30)	*p* Value
Preoperative CORTISOL (μg/dL)	Median (IQR) Min–Max	0.64 (0.52–0.95) 0.05–17.59	0.70 (0.58–2.27) 0.50–10.52	0.038
Postoperative 24 h CORTISOL (μg/dL)	Median (IQR) Min–Max	7.94 (3.07–10.74) 1.87–21.57	11.70 (7.77–17.14) 0.82–24.39	0.015

Data are presented as median (interquartile range). Between-group comparisons were performed using the Mann–Whitney U test. Cortisol values are presented in μg/dL. Postoperative cortisol comparisons were adjusted for baseline values using ANCOVA.

## Data Availability

The data sets used and/or analyzed during the current study are available from the corresponding author on reasonable request.

## Abbreviations

ASA	American Society of Anesthesiologists
BMI	Body Mass Index
Cm	Centimeter
CRP	C-Reactive Protein
ECG	Electrocardiography
ESP	Erector Spine Plane
ESPb	Erector Spine Plane Block
IV	Intravenous
HR	Heart Rate
kg	Kilogram
LDH	Lumbar Disc Herniation
mg/kg μg/dL	Milligram/kilogram Microgram/deciliter
mL	Milliliter
MTPB	Midtransverse process block
NIBP	Non-invasive blood pressure
NRS	Numeric Rating Skala
NSAID	Nonsteroidal anti-inflammatory drugs
MAP	Middle Arterial Pressure
SpO2	Peripheral Oxygen Saturation
USG	Ultrasonography
VAS	Visual Analog Scale
WBC	White Blood Cell
WHO	World Health Organization
